# Establishing a System for Medical Certification of Cause of Death for Noninstitutional Deaths in a Selected Area of Kolar District, Karnataka, India: Protocol for a Population-Based Feasibility and Validation Study

**DOI:** 10.2196/72330

**Published:** 2025-08-18

**Authors:** Madhusudan Muralidhar, Sukanya Rangamani, Vaitheeswaran Kulothungan, Priyanka Das, Monesh B Vishwakarma, Prashant Mathur

**Affiliations:** 1 ICMR-National Centre for Disease Informatics and Research Bengaluru Rural District India

**Keywords:** Medical Certification of Cause of Death, noninstitutional deaths, physician-derived cause of death, PhyCoD

## Abstract

**Background:**

Medical Certification of Cause of Death (MCCD) coverage in India is only 22.5%, largely due to a significant proportion of deaths occurring outside hospitals (noninstitutional deaths). The cause of death (CoD) in such cases is unlikely to be certified by any doctor. This study attempts to address this gap by developing an MCCD system for noninstitutional deaths in India.

**Objective:**

This study will assess the feasibility of a physician-derived cause of death (PhyCoD) approach for deducing CoD in noninstitutional deaths in a selected area of Kolar Taluk, Karnataka, and validate this approach.

**Methods:**

This population-based feasibility and validation study will be conducted in 4 selected hospitals and 2 Primary Health Centre (PHC) areas in Kolar taluk, Kolar district, Karnataka, India. We developed 4 PhyCoD questionnaires: maternal, neonatal, child, and adult. Institutional deaths that occurred over the previous 10 months in these selected hospitals with detailed case records available were selected as “gold standard” cases. Trained investigators abstracted the history from these case records into the questionnaires and deduced the CoD sequence of events. The investigators then elicited the history from the deceased’s relatives using the PhyCoD questionnaire and deduced the CoD sequence of events. This will be compared with the gold standard CoD sequence of events deduced from medical records. The extent of agreement will be measured. The tool will be revised based on the pilot phase experiences. For all brought dead cases to the 4 hospitals and home deaths in the 2 PHC areas over a 3-month period, doctors in these hospitals and PHC medical officers, respectively, will elicit the history from the deceased’s kin using the PhyCoD questionnaires and arrive at a CoD sequence of events. This CoD sequence of events will be validated against the gold standard autopsy whenever possible (in brought dead cases). The PhyCoD approach will also be tested for inter-rater reliability by independent investigators on a random sample of noninstitutional deaths.

**Results:**

Institutional ethics committee clearance (January 2024), recruitment and training of project staff (January 2024-January 2025), preparation of questionnaires and application (August 2024-February 2025), pilot phase data collection (48 cases; August 2024-December 2024), and training of the doctors in the participating hospitals and PHC medical officers (December 2024) are complete. A total of 48 cases (32 adult, 7 child, 3 maternal, and 6 neonatal) were included in the pilot phase. Data review and analysis of the pilot phase data are underway.

**Conclusions:**

The study is expected to provide information about the validity and feasibility of the PhyCoD approach. Depending on the study’s outcomes, the tool may be adopted by more states, leading to increased coverage of noninstitutional deaths under MCCD, improved accuracy, and reduced delay of CoD reporting for noninstitutional deaths.

**International Registered Report Identifier (IRRID):**

DERR1-10.2196/72330

## Introduction

**Figure 1:**
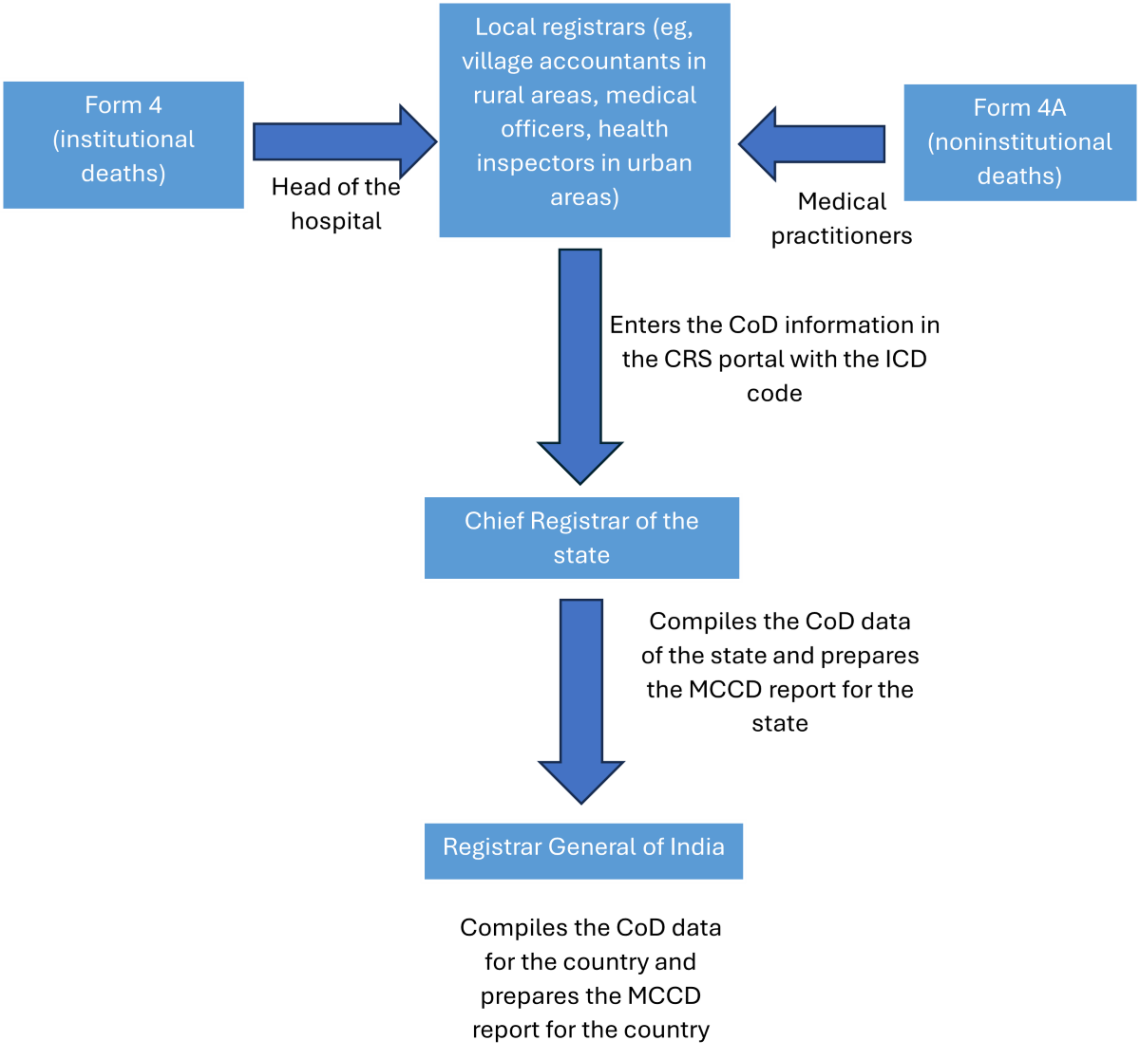
Medical Certification of Cause of Death (MCCD) data flow in India. CoD: cause of death; CRS: civil registration system; ICD: International Classification of Diseases.

Reliable age and gender-wise cause of death statistics are required on a regular basis by administrators, policy planners, and others for evidence-based decision-making regarding identifying the priority areas for resource allocation, monitoring of various indicators, and other related activities in the area of public health. Keeping this in mind, the Medical Certification of Cause of Death (MCCD) scheme was introduced in India under the provisions of the Registration of Births and Deaths (RBD) Act, 1969. The cause of death information is recorded in the prescribed formats (form 4 for institutional deaths and 4A for noninstitutional deaths) by doctors attending to the decedent during their last illness and submitted to the local registrars (Figure 1). However, the coverage of MCCD for the total number of registered deaths in India is dismal, at 22.5%, and this low coverage has hardly improved over the last decade [1]. This is largely attributed to a significant proportion of deaths (60%-70%) occurring outside the hospital (deaths at home, such as deaths during sleep and sudden deaths, and those occurring during travel to the hospital), which are classified as noninstitutional deaths. The cause of death in such cases is unlikely to be certified by any doctor.

In instances of persons declared as “brought dead” to the hospital where an unnatural cause is suspected, an autopsy is performed as per legal procedures. Even in instances where an unnatural cause is not suspected, autopsy is advised to determine the cause of death. However, the relatives of the deceased refuse an autopsy in most such cases. There are very few hospitals in India where a clinical autopsy is performed. The physician in the hospital does or can not certify the cause of death in such cases, as he/she has not treated the case. Hence, such deaths end up without a documented cause of death. A large proportion of deaths occurring in homes (eg, old age) may not even reach the hospital or a doctor at all.

The RBD Act, 1969, and its recent amendment in 2023 clearly state that, for all deaths, the cause of death has to be certified by the doctor who attended to the decedent in his/her recent illness. However, it does not clarify how the cause of death can be certified for an individual who has not received any medical care in the immediate period before death [1,2].

In recent times, most of the states in India have mandated that hospitals submit form 4 (MCCD form) for all deaths occurring in institutions in order to complete the death registration. However, for “noninstitutional deaths” (deaths occurring in any place other than medical institution), the MCCD form (form 4A) is neither insisted upon nor is any suitable guidance provided. As a result, noninstitutional deaths, which account for 60%-70% of all deaths in India, end up without a medically certified cause of death. This was one of the important observations in an implementation research study conducted in 6 states by the Indian Council of Medical Research - National Centre for Disease Informatics and Research (ICMR-NCDIR), Bengaluru. The scenario is similar across most low-income countries [3,4].

This study attempts to develop a system to address the gap in MCCD coverage for noninstitutional deaths. The recent MCCD report of India states that there is some type of contact with a medical facility just before death in 54.6% of registered deaths [1]. Hence, it may be worthwhile to establish a system for deriving the cause of death for noninstitutional deaths by involving medical doctors.

This would add to the already existent methods of arriving at an underlying cause of death: (1) MCCD for institutional deaths (under the civil registration system [CRS]) and (2) verbal autopsy (VA) under the sample registration system (SRS). VA is conducted by lay workers called SRS supervisors who collect the history from the family members of the deceased using a validated tool, and the information is subsequently reviewed by physicians to derive the cause of death (physician-coded VA). This 2-step process is time-consuming and delays assignment of the cause of death. Moreover, SRS is limited by the population coverage for deriving cause of mortality statistics at the state or national level and by the availability of doctors proficient in reviewing the narratives in many regional languages [5,6].

In this study, doctors will use a validated questionnaire to collect the history of the deceased using multiple available sources of information like relatives, medical records, and investigations to arrive at the cause of death events. As doctors are well versed in taking the history and abstracting the cause of death information to the MCCD form, this approach using a physician-derived cause of death (PhyCoD) is likely to significantly reduce time and resources in arriving at the cause of death and increase the reliability as compared with SRS VA. In this context, this study will be conducted to assess the feasibility of a PhyCoD approach for deducing the cause of death in noninstitutional deaths in a selected area of a Taluk of Kolar district, Karnataka, and to validate this approach.

## Methods

### Data Collection

This population-based feasibility and validation study will be conducted for a duration of 24 months (February 2024-January 2026) in Kolar Taluk (Taluk is an administrative subdivision of a district), Karnataka. Kolar is a district in the south Indian state of Karnataka, with a population of 15,36,401 (2011 census) and an MCCD coverage of 9.9% (much below the state level of 26.7%) [7-9]. The district is a pioneer for several health-related projects like Digital Nerve Centre of Tata Community Services and Kolar Comprehensive Primary Health Care Initiative of the Society for Community Health Awareness, Research and Action (SOCHARA) [10,11]. Four hospitals (1 government district hospital and 3 private hospitals, of which 1 is a medical college) and 2 PHC areas (1 urban and 1 rural) in Kolar Taluk have been identified to be part of the study. The identified hospitals and PHC areas are those with the highest case load and highest population, respectively, in the Taluk. ICMR-NCDIR had recently (2022-2024) conducted an implementation research study in 6 states of India. Kolar district of Karnataka was also a part of the study, and 3 of the 4 hospitals selected in this study were part of the earlier implementation research study. Hence, Kolar district was selected for the study.

**Figure 2:**
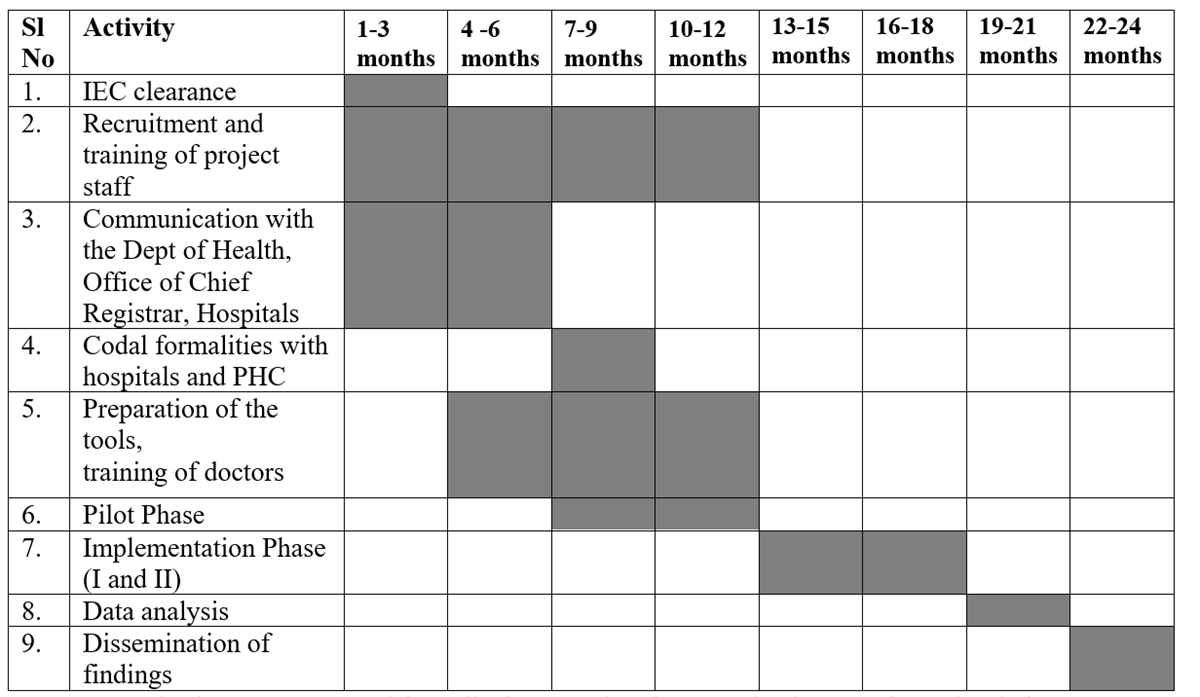
Timeline of the project. IEC: institutional ethics committee; PHC: Primary Health Centre.

The study is being implemented by the principal investigator (PI), co-PIs, and project staff from ICMR-NCDIR, Bengaluru, as per the timelines in Figure 2. Permissions have been obtained from the Department of Health and Family Welfare, Government of Karnataka, and Chief Registrar of Births and Deaths, Karnataka, and nodal officers in the district have been designated by their respective departments for further coordination. Participating hospitals and PHCs were identified based on the inputs of these nodal officers, and necessary directions were issued to these hospitals and PHCs for their cooperation.

The PhyCoD tool has been developed and comprises of 4 questionnaires, as follows: (1) questionnaire for neonatal deaths (deaths postbirth to 28 days), (2) questionnaire for child deaths (deaths 29 days onward to 14 years), (3) questionnaire for maternal deaths (for deaths of women aged 15 years to 49 years during pregnancy, delivery, or within 42 days of delivery including deaths during and within 42 days of abortion), and (4) questionnaire for adult deaths (all other deaths).

The age criteria for these questionnaires align with the SRS VA questionnaires [12]. The PhyCoD questionnaires are similar to history-taking questionnaires routinely used by doctors and comprise fields such as chief complaints, history of presenting illness, past history, examination findings, and investigations with a checklist within the questions based on the World Health Organization (WHO) and SRS VA questionnaires so as not to miss any information. Both WHO and SRS VA questionnaires contain a checklist to capture the history of the deceased, which will help the reviewing doctor arrive at the cause of death. Although the WHO VA tool has a wider focus and scope to capture a wide variety of presentations and causes of death, the ability of the SRS VA questionnaires to capture these is limited [12,13].

The chief complaints are listed in each of the PhyCoD questionnaires considering the causes of death for each of these groups as per the recent MCCD report [1]. In addition, provision has been made to capture other presentations, if any. The questionnaires also have a provision to record the narrative as given by the kin of the deceased so as to understand the sequence of events leading to death.

**Figure 3:**
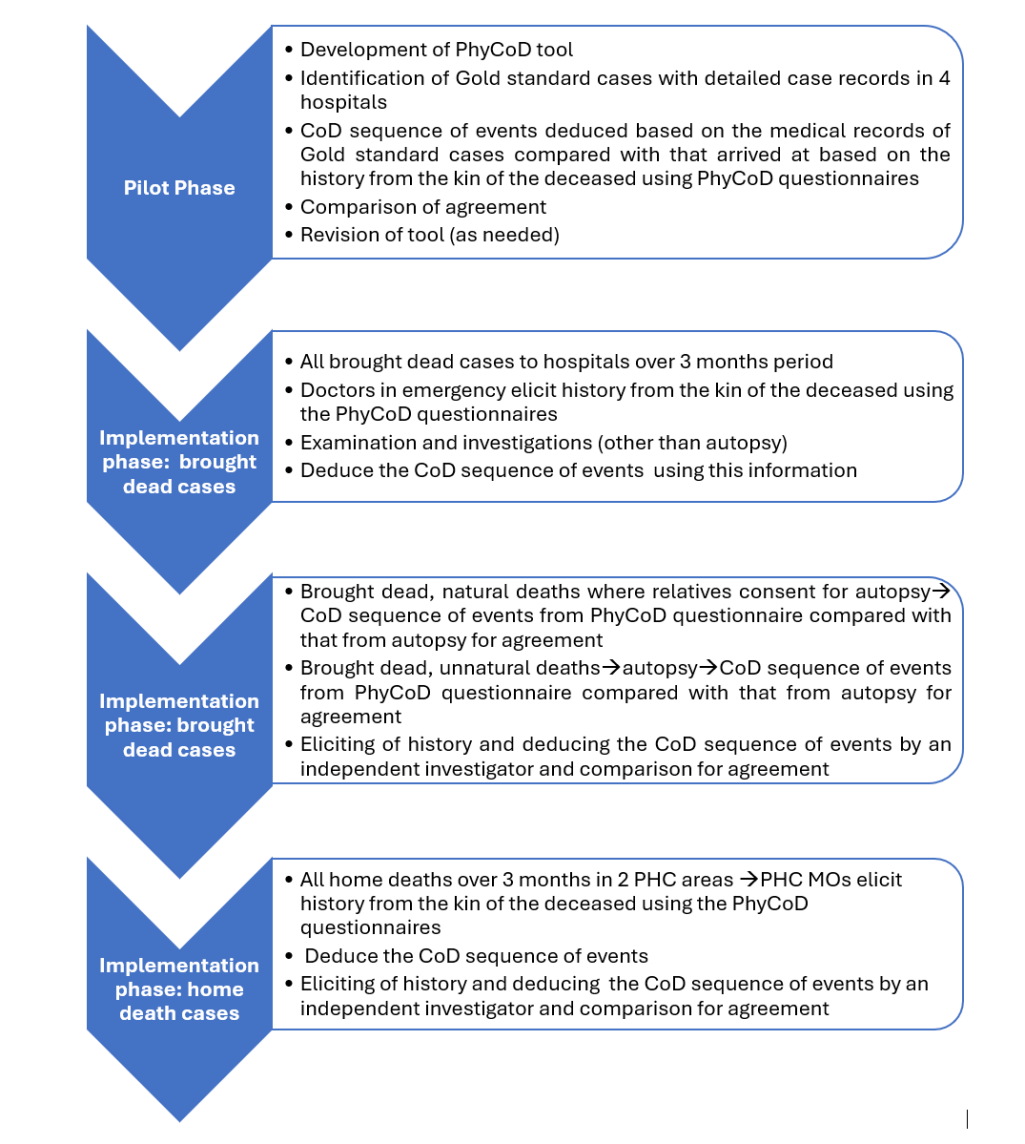
Methodology of the pilot and project phases. CoD: cause of death; MO: Medical Officer; PHC: Primary Health Centre; PhyCoD: physician-derived cause of death.

The study will be conducted in 2 phases: (1) pilot phase and (2) implementation phase (Figure 3).

During the pilot phase, institutional deaths with detailed case records that had occurred in the previous 10 months in the District Hospital and Medical College were selected. Trained investigators (who included faculty from the Community Medicine Department of the medical college and postgraduates in dental sciences and public health) and the PI abstracted the case history into the PhyCoD questionnaires and arrived at the cause of death sequence of events (ie, immediate, antecedent, and underlying) using the information. The houses of the deceased were located with the help of the district health officials and local PHC staff. These trained investigators subsequently conducted a home visit and administered the tool among relatives of the deceased to elicit the history of the deceased including a review of any available medical records and derived the cause of death sequence of events. Abstraction of the case history (at the hospital) and eliciting the history from the kin of the deceased (during the home visit) were conducted by all the investigators. However, arriving at the cause of death sequence of events was performed by medical doctors (ie, the PI and faculty from the Community Medicine Department). Blinding was ensured in such a manner that the investigator conducting the home visit for a particular case was different from the one who abstracted the case history in the hospital.

The cause of death sequence of events determined based on the history elicited from the kin of the deceased during the home visit will be compared with the “gold standard” cause of death sequence of events derived from the medical records. The agreement between the PhyCoD and the gold standard with respect to the underlying cause of death and major cause group will be measured. Based on the experiences in the initial pilot phase, the questionnaires were revised to make them more specific and user-friendly and to prevent any information from being missed. For example, new questions like treatment received for current illness were added, and specific subquestions were added within open questions like past history and antenatal, natal, and postnatal history.

The final versions of the questionnaires are available in Multimedia Appendices 1-4.

A minimum of 30 cases was selected as the sample size for the pilot phase based on the proportions of maternal, neonatal, child, and adult deaths in the CRS and SRS, as shown in Table 1 [14,15].

**Table 1:** Proportions of maternal, neonatal, child, and adult deaths in the civil registration system (CRS) and sample registration system (SRS).

Death	Proportion in CRS, %	Proportion in SRS, %	Proportion out of 30 (CRS), %	Proportion out of 30 (SRS), %
Maternal	7	6.1	2.1	1.8
Neonatal	0.2	0.5	0.1	0.1
Child	3.3	14.5	1	4.3
Adult	89.5	78.9	26.8	23.7

Accordingly, it was decided to have at least 3 maternal, 2 neonatal, 5 child, and 27 adult cases. In addition, for each type of death, an attempt was made to include the different types of cause of death within the category (eg, communicable diseases, noncommunicable diseases, external cause for adult) subject to availability.

The pilot phase has been completed, and data review and analysis are underway.

In the implementation phase, the tool will be administered in 2 settings: (1) brought dead cases to the 4 hospitals and (2) home deaths in the 2 selected PHC areas.

The process of handling the brought dead cases in the hospital and the roles and responsibilities of various stakeholders will be mapped. Similarly, the process of reporting, death registration, and MCCD in home death cases will be mapped. At least 1 doctor from the major departments (where deaths occur) in each of the 4 hospitals and the Community Medicine Department (in case of the medical college) and medical officers from the 2 PHC areas have been trained in MCCD, collecting history using the PhyCoD tool, and arriving at cause of death sequence of events using this information in noninstitutional deaths. All noninstitutional deaths (deaths at home and during travel to the hospital or clinic, including unnatural deaths) that occur over a 3-month period will be considered. Post hoc power analysis will be done at the end of the study to determine the power.

Brought dead cases to the 4 hospitals over a period of 3 months starting from January 1, 2025, will be considered. Doctors in casualty or assigned for the research study will elicit the history from the kin of the deceased using the PhyCoD questionnaire including a review of available medical records. They will also perform the examinations (other than autopsy) and investigations that are relevant for arriving at the cause of death and will record all these findings in the PhyCoD questionnaire [16]. Based on all this information, the doctors will arrive at the cause of death sequence of events.

All brought dead cases that are suspected to be unnatural will undergo an autopsy as per the rules. In cases of brought dead cases that are likely to be natural deaths, the attending doctors will counsel the relatives about the need for a clinical autopsy to determine the cause of death for public health purposes. In the instances where the relatives agree, a clinical autopsy will be performed. The PhyCoD sequence of events will be compared with that from the autopsy report to determine the extent of agreement.

History will be collected from one or more attendants accompanying the deceased. They may include relatives, friends, or acquaintances of the deceased who lived with the deceased or know the medical history of the deceased as well as eyewitnesses like co-passengers, passersby, and police at the time of death.

Deaths occurring over a period of 3 months (starting from December 24, 2024) at home in the 2 PHC areas will be considered. Information on deaths will be obtained from health workers of the PHC and District Statistical Office.

The PHC medical officers of the 2 PHC areas will visit the houses of the deceased after the grieving period but within 1 month of death and elicit the history using the PhyCoD tool from the relatives, review the medical records if any, and deduce the cause of death sequence of events. Community Health Officers (nursing graduates) in the PHC area have also been trained to elicit a history using the PhyCoD questionnaires to complement the doctors in the event of the PHC Medical Officer being unable to cover all home deaths. However, deducing the cause of death sequence of events will be done by the PHC Medical Officer only, based on the review of history collected using the PhyCoD questionnaire.

History will be collected from one or more informants, who may include relatives, friends, or acquaintances of the deceased who stay with the deceased or know the medical history of the deceased as well as eyewitnesses at the time of death.

Data collection for the project phase started on January 1, 2025, and is expected to be completed by May 31, 2025.

### Validation

In brought dead cases for which an autopsy is performed, the PhyCoD sequence of events will be compared with that from the autopsy report (gold standard) for accuracy.

In a 20% sample of home deaths in the 2 PHC areas and a 10% sample of brought dead cases (other than those for which autopsy is done) in 4 hospitals selected by simple random sampling, an independent trained physician will elicit the history from the kin of the deceased using the PhyCoD tool and deduce the cause of death sequence of events. The extent of agreement between the cause of death sequence of events deduced by the 2 physicians will be measured (inter-rater reliability).

### Data Collection, Software Development, and Database Management

A web-based application has been developed to capture information pertaining to neonatal, child, adult, and maternal deaths using the latest Microsoft technologies and MS SQL Server. The questionnaires have skip patterns, validations, and built-in quality checks (eg, for all female deaths, the pregnancy and delivery-related field in the MCCD form is mandatory; for all deaths for which autopsy field is completed in the questionnaire, a mandatory second MCCD form is enabled to be completed by the doctor conducting the autopsy) to capture accurate information. The application has access-based privileges for data entry, searching records, and downloading records. The web-based application allows access from any location with an internet connection, and the data will be synced directly with the online database server hosted by ICMR-NCDIR.

Data will be collected using tabs by the doctors of the 4 participating hospitals and 2 PHC Medical Officers. All data collected will be stored on a secure server at ICMR-NCDIR for subsequent retrieval and analysis.

### Statistical Analysis

The kappa statistic will be used to measure extent of agreement between the cause of death sequence of events (1) deduced from the case records and that arrived at using the PhyCoD questionnaire from the kin of the deceased during the home visit (pilot phase), (2) deduced by the physician and that deduced by autopsy (implementation phase), and (3) deduced by doctors at the participating hospitals and PHC Medical Officers and that by independent assessors (implementation phase)

The extent of coverage of brought dead cases in the 4 hospitals and home deaths in the 2 PHC areas for eliciting the history using the PhyCoD questionnaire will be measured. The challenges encountered by various stakeholders during implementation will also be recorded.

### Ethical Considerations

Ethical clearance has been obtained from the ICMR-NCDIR Institutional Ethics Committee (number NCDIR/IEC/3073/2023, 21/02/2024). Written informed consent will be obtained from the kin of the deceased in the presence of impartial witness(es). Identity of the deceased and contact details of the kin of the deceased will be collected for the purpose of clarification, if any, pertaining to the history at a later stage. However, these will be kept strictly confidential and will not be shared with any unauthorized third party or revealed in any publications. Data will be stored in password-protected systems, and only the PI and co-PIs will have access.

## Results

The following activities have been completed (Figure 2): institutional ethics committee clearance (January 2024), recruitment and training of project staff (January 2024-January 2025), preparation of questionnaires and the web application (August 2024-February 2025), pilot phase data collection (48 cases; August 2024-December 2024), and training of center PIs, doctors at the 4 participating hospitals, and 2 PHC Medical Officers (December 2024).

Although a total of 60 cases were selected for the pilot phase, 12 were not reachable for home visits. Hence, a total of 48 cases (32 adult, 7 child, 3 maternal, and 6 neonatal) were included in the pilot phase. Data review and analysis of the pilot phase data are underway.

## Discussion

This study intends to address the gap in coverage as well as accuracy of MCCD for noninstitutional deaths in India, which account for more than 60% of the registered deaths. The study intends to develop a tool for physicians to deduce the cause of death for noninstitutional deaths. The study will also describe the feasibility of completing the MCCD for noninstitutional deaths using existing manpower and infrastructure as well as the likely challenges in achieving this. It will also validate the approach. 

The findings of the study shall be shared with the Office of the Registrar General of India, Chief Registrar of Births and Deaths of Karnataka, and the Department of Health, Karnataka. Based on the outcomes of the project, the approach may be subsequently implemented in other districts and states of the country to increase the coverage of MCCD for noninstitutional deaths.

The likely challenges that may be encountered during the implementation of the project are described in the following paragraphs. There may be very limited history available in a significant proportion of cases such as sudden death in healthy middle-aged persons with no other previous illnesses and frail older adults (>80 years) presenting with only generalized weakness and no other morbidities.

In such cases, the doctors would have to rely on investigations and clinical autopsy, which may be helpful in arriving at the cause of death. These may be refused by the kin of the deceased in a significant proportion of cases.

In a certain proportion of cases, there will be limited information available in the medical records or from the kin of the deceased. This would pose an important challenge in validating the questionnaires during the pilot phase. In addition, in a significant proportion of cases, especially individuals with multiple morbidities, it could be difficult to delineate the cause of death sequence of events. This could be an important challenge during the implementation phase.

The strength of the study is that it attempts to leverage the experience and skills of medical doctors from within the health system for increasing the coverage of MCCD for noninstitutional deaths. Studies in the past have only attempted to strengthen the quality of VA under SRS. The limitation of VA is that history is collected by a lay person (SRS supervisor) from the kin of the deceased and subsequently reviewed by a medical doctor (who is located far away is not aware of the context) to arrive at cause of death. Because of this, there are chances of information being lost or misinterpreted. Also, the history collected in VA under SRS is in the local language. Hence, a considerable number of medical doctors proficient in various regional languages need to be available. This is a challenge in the case of some regional languages. In addition, because of the complicated process, there is a considerable delay in arriving at cause of death (approximately 9 months) [5,17]. These limitations are likely to be addressed by this approach. The medical knowledge of the doctors may significantly augment the quality of history elicited. In addition, the doctor has an opportunity to perform a physical examination of the body in brought dead cases, which is also likely to add to the accuracy of the derived cause of death.

## Appendix

Multimedia Appendix 1 Maternal death questionnaire.

Multimedia Appendix 2 Adult death questionnaire.

Multimedia Appendix 3 Neonatal death questionnaire.

Multimedia Appendix 4 Child death questionnaire.

## Abbreviations

CRS: civil registration system
ICMR: Indian Council of Medical Research
ICMR-NCDIR: ICMR-National Centre for Disease Informatics and Research
MCCD: Medical Certification of Cause of Death
PHC: Primary Health Centre
PhyCoD: physician-derived cause of death
PI: principal investigator
RBD: Registration of Births and Deaths
SOCHARA: Society for Community Health Awareness, Research and Action
SRS: sample registration system
VA: verbal autopsy
WHO: World Health Organization

## Data Availability

The data will be deposited into the institutional data repository of the Indian Council of Medical Research-National Centre for Disease Informatics and Research (ICMR-NCDIR), and access to other researchers will be provided subject to approval of the competent authority.
