# Supplementary material for: Establishing a System for Medical Certification of Cause of Death for Noninstitutional Deaths in a Selected Area of Kolar District, Karnataka, India: Protocol for a Population-Based Feasibility and Validation Study
Source: JMIR Res Protoc. 2025 Aug 18;14:e72330. doi: 10.2196/72330 (PMC12402730; doi:10.2196/72330)
Supplement: Multimedia Appendix 1 [file resprot_v14i1e72330_app1.pdf]

Setting up of a system for Medical Certification of Cause of Death for non-institutional deaths in a selected area of a Taluk of Kolar district, Karnataka: feasibility and validity

Maternal Death Questionnaire (15-49 yrs)

I. General Details

Date of the Interview:

Serial No:	
Name of the doctor collecting history	
Name of the Deceased	
Name of the Husband/Father	
Age of the deceased (in years)	
House address:	
Date of death	
Time of death	Not Available ☐
Place of death	Home ☐<br>Others ☐<br>If others Specify below<br>☐
Name of the Hospital where treated/Brought dead	

II. Period of death:(for maternal death tick the box applicable below)

- ☐ During pregnancy: Trimester _____week ___________
- ☐ During labour or within 1 hr of delivery
- ☐ From > 1 hr of delivery and 6 weeks of delivery
- ☐ During abortion or within 6 weeks of abortion

III. Respondent details

Sl No	Name	Relationship	Contact No
1.			
2.			
3.			

IV. Chief Complaints and Duration

(Based on the selection of chief complaints, duration box will appear for that specific chief complaint)

Sl No	Complaint	**Duration** (specify in days or hours**)**
1.	Fever	
2.	Malaise	
3.	Pallor	
4.	Yellowish discoloration of eyes, palms, nail beds	
5.	Convulsions	
6.	Vomiting	
7.	Edema/swelling	
8.	Vaginal bleeding	
9.	Discharge from vagina	
10.	Amenorrhea	
11.	Bleeding from the mucosa	
12.	Headache	
13.	Blurring of vision	
14.	Aura	
15.	Loss of consciousness	
16.	Abdominal pain/tenderness	
18.	Chest pain	
19.	Breathing difficulty	
20.	Palpitation	
21.	Excessive weight gain in pregnancy	
22.	Low weight gain in pregnancy	
23.	Dark colored urine	
24.	Retention of urine	
25.	Incontinence of Urine	
26.	Cracked & painful nipples	
27.	Painful breast	
28.	Congenital Malformation of any part	
29.	Any external causes (like poisoning, road traffic accidents, etc.,) *<br>Yes ☐<br>No ☐	
30.	Others specify	

**31.**	**Others specify**	
**32.**	**Others specify**	

a) *mandatory

b) if no other chief complaint is chosen then, at least one "others specify" shall be mandatorily recorded.

V. History of Presenting Illness

(Based on the selection of chief complaints, details box will appear for that specific chief complaint)

Sl No	Complaint	Details
**1.**	**Fever**	Elaborate on nature, severity, aggravating, relieving factors, associated complaints
**2.**	**Malaise**	Elaborate on the severity
**3.**	**Pallor**	Elaborate on the site, severity, associated complaints
**4.**	**Yellowish discoloration of eyes, palms, nail beds**	Elaborate on the site, severity and associated complaints
**5.**	**Convulsions**	Elaborate on the parts of the body affected, number and duration of episodes, precipitating/aggravating, relieving factors, characteristics, associated complaints
**6.**	**Vomiting**	Elaborate on the number of episodes, time of the day, nature, content of the vomitus, precipitating factors, associated complaints
**7.**	**Edema/swelling**	Elaborate on number of episodes, duration of episodes, region involved, severity, nature, precipitating factors, progress
**8.**	**Vaginal bleeding**	Elaborate on severity, number and duration of episodes, precipitating factors
**9.**	**Discharge from vagina**	Elaborate on the characteristics of discharge, number and duration of episodes, associated complaints
**10.**	**Amenorrhea**	Elaborate on the associated complaints
**11.**	**Bleeding from the mucosa**	Elaborate on the sites, severity, size, number of episodes, quantity of blood lost in each episode, precipitating factor(s)
**12.**	**Headache**	Elaborate on the nature, severity, precipitating factors, aggravating and relieving factors, associated complaints

**13.**	**Blurring of vision**	Elaborate on the precipitating factor, number of episodes, duration of episodes, associated complaints
**14.**	**Aura**	Elaborate on the characteristics, precipitating factors, number of episodes, duration of episodes, associated complaints
**15.**	**Loss of consciousness**	Elaborate on the no of episodes, duration of episodes, precipitating factors, associated complaints
**16.**	**Abdominal pain/tenderness**	Elaborate on the site, number of episodes, duration of episodes, precipitating, aggravating and relieving factors, severity, characteristics, radiation, progression
**18.**	**Chest pain**	Elaborate on the number of episodes, duration of episodes, precipitating, aggravating and relieving factors, severity, characteristics, radiation, progression
**19.**	**Breathing Difficulty**	Elaborate on the nature, associated complaints (intercostal suction, abnormal sounds produced), precipitating, aggravating and relieving factors
**20.**	**Palpitation**	Elaborate on severity, number of episodes, duration of episodes, associated complaints
**21.**	**Excessive weight gain in pregnancy**	Elaborate on the amount of weight gained over the entire gestation period or during each trimester
**22.**	**Low weight gain in pregnancy**	Elaborate on the amount of weight gained over the entire gestation period or during each trimester
**23.**	**Dark colored urine**	Elaborate on the color, amount, frequency and associated complaints
**24.**	**Retention of urine**	Elaborate on severity, precipitating factors, progress
**25.**	**Incontinence of Urine**	Elaborate on nature, severity, precipitating factors
**26.**	**Cracked & painful nipples**	Elaborate on the severity, unilateral/bilateral, precipitating factors, associated complaints
**27.**	**Painful breasts**	Elaborate on the severity, site, precipitating factors

**28.**	**Congenital Malformation of any part**	Specify site, nature
**29.**	**Any external causes (like poisoning, road traffic accidents, etc.,) ***<br>**Yes ☐ (If Yes elaborate on this)**<br>**No ☐**	Elaborate on the circumstances, intent (accident, suicide, homicide etc.,), site of injury and place of occurrence
**30.**	**Others Specify**	Elaborate on the site, number of episodes, duration of episodes, severity, nature, precipitating factors, relieving factors, associated complaints
**31.**	**Others Specify**	Elaborate on the site, number of episodes, duration of episodes, severity, nature, precipitating factors, relieving factors, associated complaints
**32.**	**Others Specify**	Elaborate on the site, number of episodes, duration of episodes, severity, nature, precipitating factors, relieving factors, associated complaints

a) *mandatory

b) If no other chief complaint is chosen then, at least one “others specify “Shall be made mandatorily recorded

VI. Treatment received for the current illness

(Record the history of any treatment received for the current illness)

....

....

Nil ☐

VII. Present obstetric history

a) Antenatal history

Obstetric score	☐	Not Available	☐
LMP	☐	Not Available	☐
EDD	☐	Not Available	☐
No. of ANC Checkups	☐	Not Available	☐
Were IFA tablets taken?	☐	Not Available	☐
Was TT taken	☐	Not Available	☐
Bleeding per vagina	☐	Not Available	☐
H/o high B.P	☐	Not Available	☐
H/o anemia during pregnancy	☐	Not Available	☐

H/o sharp abdominal pain		Not Available	☐
Giddiness/fainting		Not Available	☐
Any other diseases/significant events		Not Available	☐

b) Natal history (if applicable)

(Ask if the delivery happened or not. If Yes, record natal history of the current pregnancy, If No, record abortion history of the current pregnancy. Record the response/details in the box provided. Respond only if ''2,'3' or '4' is chosen Sl No II)

Was there a delivery? Yes ☐ No ☐

If there was a delivery:

Date of delivery		Not Available	☐
No.of babies delivered		Not Available	☐
Mode of delivery		Not Available	☐
Reason		Not Available	☐
Place of delivery		Not Available	☐
Person conducting delivery		Not Available	☐
Any significant events during delivery		Not Available	☐

If there was an abortion (Respond only if '4' is chosen Sl No II)

Date of abortion		Not Available	☐
No. of fetus aborted		Not Available	☐
Type of abortion		Not Available	☐
Reason		Not Available	☐
Place of abortion		Not Available	☐
Person conducting abortion		Not Available	☐
Any significant events during abortion		Not Available	☐

c) Post Natal history (till 6 weeks post delivery, if applicable) (Record the response/details in the box provided. Respond only if '3' or '4' is chaosen Sl No II)

Bleeding per vagina		Not Available	☐
Fever		Not Available	☐
Any major surgical procedure		Not Available	☐
Any other significant events post delivery		Not Available	☐

VIII. Past obstetric history (if applicable)

(Record the response/details in the box provided)

Number of pregnancies		Not Available	☐
Mode(s) of delivery		Not Available	☐
Reason		Not Available	☐
Any other significant events during pregnancy, delivery or post-natal period		Not Available	☐

IX. Past History

(Please record duration since diagnosis and treatment received for all the selected diseases. If your selection is "Major Surgeries" then please specify the type of surgery and also the duration since the surgery)

Diabetic Mellitus	Elaborate on duration and treatment taken
Hypertension	Elaborate on duration and treatment taken
Tuberculosis	Elaborate on duration and treatment taken
Epilepsy	Elaborate on duration and treatment taken
Bronchial asthma	Elaborate on duration and treatment taken
Covid-19	Elaborate on duration and treatment taken
Others	Elaborate on duration and treatment taken
Major surgeries	Specify the type and the duration since the surgery

X. Family History

(Similar illnesses, any other communicable diseases, consanguineous marriage, psychiatric illness, tobacco/alcohol/drug abuse in the family)

....

....

Nil Significant ☐

XI. Epidemiological History

(History of recent travel, contact with similar cases (for communicable diseases), etc.)

....

....

Nil Significant ☐

XII. Socio-environmental History

(Overcrowding, lack of ventilation, source of drinking water, water purification methods used, etc.)

....

....

Nil Significant ☐

XIII. Personal history

(Record any personal history available for the deceased as per the given field)

a) Bowel

Not available ☐ N.A.D ☐

b) Bladder

Not Available ☐ N.A.D ☐

c) Sleep

Not Available ☐ N.A.D ☐

d) Appetite

Not Available ☐ N.A.D ☐

e) Addictions

(Elaborate on the duration and nature)

Not Available ☐ Nil ☐

XIV. General Physical Examination (Record the response/details in the box provided)

External causes

Clothing

(Any evidence of violence, wetness, burns, any foreign substance)

Nil Significant ☐

Entire body

(Any evidence of injuries; describe the site, number, and nature of injuries; any foreign substance or bodily fluids/discharge)

Nil significant ☐

Eyes

(Any hemorrhage)

Nil Significant ☐

Ears

(Any hemorrhage, CSF in the canal)

Nil Significant ☐

Mouth

(Evidence of any foreign substance)

Nil Significant ☐

Routine

Pallor

(Elaborate on the site and severity)

Nil Significant ☐

Icterus

(Elaborate on the site and severity)

Nil Significant ☐

Cyanosis

(Elaborate on the site, severity, characteristics)

Nil Significant ☐

Clubbing

(Elaborate on the grade)

Nil Significant ☐

Lymphadenopathy

(Elaborate on distribution (Localized/generalized), site, characteristics)

Nil Significant ☐

Loss of subcutaneous fat

(Elaborate on distribution, severity)

Nil Significant ☐

Edema

(Elaborate on region involved, severity, nature)

Nil Significant ☐

Signs of Dehydration

(Elaborate on region involved, severity)

Nil Significant ☐

Vitals

**BP**	in mmHg	Not Available	☐
**Pulse**	in bpm	Not Available	☐
**RR**	in cycles/min	Not Available	☐
**Temperature**	in °F	Not Available	☐

XV. Systemic examination (Record the response/details in the box provided)

a) RS

Not Available ☐ N.A.D ☐

b) **CVS**

Not Available ☐ N.A.D ☐

c) **P/A**

Not Available ☐ N.A.D ☐

d) **CNS**

Not Available ☐ N.A.D ☐

XVI. Investigations

(List the findings from relevant investigations that have been conducted. Additionally, order any other investigations that you believe are necessary to determine the cause of death.)

Haematological investigations

Not Available ☐ N.A.D ☐

Renal Function Tests

Not Available ☐ N.A.D ☐

Liver Function Tests

Not Available ☐ N.A.D ☐

Serum Lipid Profile

Not Available ☐ N.A.D ☐

Radiological Investigations

Not Available ☐ N.A.D ☐

Others

Not Available ☐ N.A.D ☐

Autopsy Findings

If Autopsy is done for the case, then Form 4 in serial no. XX also needs to be filled

Not Available ☐ N.A.D ☐

XVII. Remarks/Narrative

(Please record the diagnosis, sequence of events from MCCD form if available, any other information and narrative from the kin of the deceased)

XVIII. Summary

(All responses recorded above will auto populate in the respective fields except those recorded as NAD, Nil significant or Not available)

**Serial No**	
**Name of the doctor collecting history**	
**Name of the Deceased**	
**Name of the Husband/Father**	
**Age of the deceased**	
**House address**	
**Date of death**	
**Place of death**	
**Period of death**	
**Chief Complaints and Duration**	
**History of Presenting Illness**	
**Treatment received for current illness**	
**Present obstetric history**<br>a) Antenatal History<br>b) Natal History<br>c) Post Natal History	
**Past obstetric history**	
**Past medical history**	
**Family History**	
**Epidemiological History**	
**Socio-environmental History**	
**Personal history**	
**General Physical Examination**	
**Systemic examination**	
**Investigations**	
**Remarks/ Narrative/additional information if any**	

XIX. FORM NO. 4 (To be completed by the Clinician attending to the case)

FORM NO. 4

(See Rule 7)

MEDICAL CERTIFICATE OF CAUSE OF DEATH

(Hospital in-patients. Not to be used for still births)

To be sent to Registrar along with Form No.2 (Death Report)

Name of the Hospital...

I hereby certify that the person whose particulars are given below died in the hospital in Ward No...............on.....................................

at............A.M./P.M.

Name of the Deceased					For use of Statistical Office
Sex	Age at Death				
	If 1 year or more, age in Years	If less than 1 year, age in Months	If less than one month, age in Days	If less than one day, age in Hours	
1. Male<br>2. Female					
**CAUSE OF DEATH**				Interval between on set & death approx	
I					
Immediate Cause	(a)				
State the disease, injury or complication which caused death, not the mode of dying such as heart failure, asthenia, etc.	Due to (or as a consequences of)			....	....
Antecedent Cause	(b)			....	....
Morbid conditions, if any, giving rise to the above Cause, stating underlying conditions last	Due to (or as a consequences of)<br>(c)			....	....
II					
Other significant conditions contributing to the death but not related to the disease or conditions causing it	....<br>...			....................<br>....................	....................<br>....................

Manner of death

1. Natural 2. Accident 3. Suicide 4.Homicide

5. Pending investigation

How did the injury occur?

If deceased was a female, was pregnancy death associated with? 1. Yes 2. No

If yes, was there a delivery? 1.Yes 2.No.

Name and signature of the Medical Attendant certifying the cause of death

Date of verification...

XX. FORM NO.4 (To be completed by the person performing the autopsy)

FORM NO. 4

(See Rule 7)

MEDICAL CERTIFICATE OF CAUSE OF DEATH

(Hospital in-patients. Not to be used for still births)

To be sent to Registrar along with Form No.2 (Death Report)

Name of the Hospital...

I hereby certify that the person whose particulars are given below died in the hospital in Ward No...............on.................................

at............A.M./P.M.

Name of the Deceased					For use of Statistical Office
Sex	Age at Death				
	If 1 year or more,age in Years	If less than 1 year, age in Months	If less than one month, age in Days	If less than one day, age in Hours	
1. Male<br>2. Female					

CAUSE OF DEATH		Interval between on set & death approx	
I			
Immediate Cause	(a)		
State the disease, injury or complication which caused death, not the mode of dying such as heart failure, asthenia, etc.	Due to (or as a consequences of)	....	....
Antecedent Cause	(b)	....	....
Morbid conditions, if any, giving rise to the above Cause, stating underlying conditions last	Due to (or as a consequences of)<br>(c)	....	....
II			
Other significant conditions contributing to the death but not related to the disease or conditions causing it	....<br>...	..........................<br>..........................	....................<br>....................

<u>Manner of death</u>

1. Natural 2. Accident 3. Suicide 4.Homicide

5. Pending investigation

How did the injury occur?

If deceased was a female, was pregnancy death associated with? 1. Yes 2. No

If yes, was there a delivery? 1.Yes 2.No.

Name and signature of the Medical Attendant certifying the cause of death

Date of verification...
