# Supplementary material for: Establishing a System for Medical Certification of Cause of Death for Noninstitutional Deaths in a Selected Area of Kolar District, Karnataka, India: Protocol for a Population-Based Feasibility and Validation Study
Source: JMIR Res Protoc. 2025 Aug 18;14:e72330. doi: 10.2196/72330 (PMC12402730; doi:10.2196/72330)
Supplement: Multimedia Appendix 2 [file resprot_v14i1e72330_app2.pdf]

Setting up of a system for Medical Certification of Cause of Death for non-institutional deaths in a selected area of a Taluk of Kolar district, Karnataka: feasibility and validity

Adult Death Questionnaire (15 years and above)

I. General Details

Date of the Interview:

Serial No	
Name of the doctor collecting history	
Name of the Deceased	
Name of the Husband/Father of the deceased	
Sex of the deceased	
Age of the deceased (in years)	
House address	
Occupation of the deceased	
Date of death	
Time of death	Not Available ☐
Place of death	Home ☐<br>Others ☐<br>If others Specify below<br>☐
Name of the Hospital where treated/brought dead	

II. Respondent details

Sl No	Name	Relationship	Contact No
1.			
2.			
3.			

III. Chief Complaints and Duration

(Based on the selection of chief complaints, duration box will appear for that specific chief complaint)

Sl No	Complaint	Duration (specify in days and if < 1 day in hours)
1.	Fever	
2.	Cough	

**3.**	**Blood in sputum**	
**4.**	**Breathing difficulty**	
**5.**	**Easy fatigability**	
**6.**	**Palpitations**	
**7.**	**Chest Pain**	
**8.**	**Chest Tightness**	
**9.**	**Pain Abdomen**	
**10.**	**Loose stools**	
**11.**	**Blood in stools**	
**12.**	**Mucus in stools**	
**13.**	**Vomiting**	
**14.**	**Blood in vomitus**	
**15.**	**Yellowish discoloration of eyes, palms, nailbeds**	
**16.**	**Decreased passage of urine**	
**17.**	**Dark colored urine**	
**18.**	**Weakness of limbs/ Muscular Weakness**	
**19.**	**Generalized weakness**	
**20.**	**Weight loss**	
**21.**	**Loss of sensation**	
**22.**	**Abnormal sensations**	
**23.**	**Lethargy/excessive drowsiness**	
**24.**	**Headache**	
**25.**	**Dizziness**	
**26.**	**Convulsions**	
**27.**	**Loss of consciousness**	
**28.**	**Altered sensorium**	
**29.**	**Visual Changes**	
**30.**	**Swelling (Edema)**	
**31.**	**Wounds**	
**32.**	**Burns**	
**33.**	**Cold extremities**	
**34.**	**Skin Rashes**	
**35.**	**Bleeding from the skin**	
**36.**	**Bleeding from the mucosa**	
**37.**	**Lumps**	
**38.**	**Any external causes (like poisoning, road traffic accidents, etc.,) ***<br>**Yes** ☐<br>**No** ☐	
**39.**	**Others specify**	
**40.**	**Others specify**	
**41.**	**Others specify**	

a) *mandatory

b) if no other chief complaint is chosen then, at least one “others specify” shall be mandatorily recorded.

IV. History of Presenting Illness

(Based on the selection of chief complaints, details box will appear for that specific chief complaint)

Sl No	Complaint	Details
1.	**Fever**	Elaborate on nature, severity, aggravating, relieving factors, associated complaints
2.	**Cough**	Elaborate on the severity, frequency of Cough, nature, sputum characteristics, aggravating, relieving factors, associated complaints
3.	**Blood in sputum**	Elaborate on the number of episodes, quantity of blood in sputum, associated complaints
4.	**Breathing Difficulty**	Elaborate on the associated manifestations (intercostal suction, abnormal sounds produced), associated complaints
5.	**Easy fatigability**	Elaborate on the number and duration of episodes, severity, progress and associated complaints
6.	**Palpitations**	Elaborate on the number and duration of episodes, precipitating factor(s) and associated complaints
7.	**Chest Pain**	Elaborate on the number of episodes, duration of episodes, severity, characteristics, radiation, associated complaints
8.	**Chest Tightness**	Elaborate on the severity, number and duration of episodes, site, precipitating, aggravating, relieving factors, associated complaints
9.	**Pain Abdomen**	Elaborate on the site, radiation, nature of pain, precipitating, aggravating and relieving factors, progress, associated complaints
10.	**Loose stools**	Elaborate on the number of episodes, consistency, color, contents, smell, associated complaints
11.	**Blood in stools**	Elaborate on the number of episodes, quantity of blood lost in each episode, precipitating factors, associated complaints

12.	**Mucus in stools**	Elaborate on the number of episodes, quantity of mucus lost in each episode, precipitating factors, associated complaints
13.	**Vomiting**	Elaborate on the number of episodes, nature, content of the vomitus, precipitating factors, associated complaints
14.	**Blood in vomitus**	Elaborate on the number of episodes, quantity of blood in vomitus, precipitating factors, associated complaints
15.	**Yellowish discoloration of eyes, palms, nailbeds**	Elaborate on the site, severity, and associated complaints
16.	**Decreased passage of urine**	Elaborate on the amount, frequency, and associated complaints
17.	**Dark colored urine**	Elaborate on the color, amount, frequency and associated complaints
18.	**Weakness of limbs/ Muscular Weakness**	Elaborate on the site, precipitating factor(s), progress, associated complaints
19.	**Generalized weakness**	Elaborate on the severity, number and duration of episodes, associated complaints, progress
20.	**Weight loss**	Elaborate on the loss of weight over the duration, associated complaints, etc.,
21.	**Loss of sensation(s)**	Elaborate on the site, sensations lost, severity
22.	**Abnormal sensations**	Elaborate on the site, type of sensation(s), number and duration of episodes
23.	**Lethargy/excessive drowsiness**	Elaborate on the precipitating factor(s), relieving factors, number and duration of episodes
24.	**Headache**	Elaborate on the nature, severity, precipitating, aggravating, and relieving factors, associated complaints

25.	**Dizziness**	Elaborate on the severity, number of episodes, duration of episodes, precipitating and relieving factors, associated complaints
26.	**Convulsions**	Elaborate on the nature, parts of the body affected, number of episodes, precipitating, aggravating, relieving factors, associated complaints
27.	**Loss of consciousness**	Elaborate on the number and duration of episodes, precipitating factors, progress, associated complaints
28.	**Altered sensorium**	Elaborate on the number and duration of episodes, severity and nature (e.g., dementia, hallucinations, confusion, disorientation etc.,), precipitating factor(s), progress and associated complaints
29.	**Visual Changes**	Elaborate on the number and duration of episodes, nature (blurring, double vision, aura), precipitating and relieving factors, associated complaints
30.	**Swelling (Edema)**	Elaborate on region involved, severity, characteristics, precipitating, aggravating and relieving factors, progress, associated complaints
31.	**Wounds**	Elaborate on the site, extent, number, nature, precipitating factor, progress, associated complaints
32.	**Burns**	Elaborate on the site, extent, number, nature, precipitating factor, progress, associated complaints
33.	**Cold extremities**	Elaborate on the severity, site, number of episodes, duration of episodes and associated complaints
34.	**Skin Rashes**	Elaborate on the number/density, characteristics, distribution, evolution and associated complaints
35.	**Bleeding from the skin**	Elaborate on the site, severity, size, number, precipitating factor(s), associated complaints

36.	**Bleeding from the mucosa**	Elaborate on the site, severity, size, number, precipitating factor(s), associated complaints
37.	**Lumps**	Elaborate on the site, size, characteristics, precipitating factor(s), progress, associated complaints
38.	**Any external causes (like poisoning, road traffic accidents, etc.,)*** **Yes** ☐ **If Yes, elaborate in the details box beside** **No** ☐	Elaborate on the circumstances of the event, intent (accident, suicide, homicide, etc.,), nature, site of injury, and place of occurrence
39.	**Others specify**	Elaborate on the site, number of episodes, duration of episodes, severity, nature, precipitating factors, relieving factors, associated complaints
40.	**Others specify**	Elaborate on the site, number of episodes, duration of episodes, severity, nature, precipitating factors, relieving factors, associated complaints
41.	**Others specify**	Elaborate on the site, number of episodes, duration of episodes, severity, nature, precipitating factors, relieving factors, associated complaints

a) *mandatory

b) If no other chief complaint is chosen then, at least one “others specify “ shall be mandatorily recorded

V. Treatment received for the current illness

(Record the history of any treatment received for the current illness)

....

....

Nil ☐

VI. Past History

(Please record duration since diagnosis and treatment taken for all the selected diseases. If your selection is “Major Surgeries” then please specify the type of surgery and also the duration since the surgery)

Diabetic Mellitus	Elaborate on duration and treatment taken

Hypertension	Elaborate on duration and treatment taken
Tuberculosis	Elaborate on duration and treatment taken
Epilepsy	Elaborate on duration and treatment taken
Bronchial asthma	Elaborate on duration and treatment taken
Covid-19	Elaborate on duration and treatment taken
Others	Elaborate on duration and treatment taken
Major surgeries	Specify the type of surgery

VII. Family History

(Similar illnesses, any other communicable diseases, consanguineous marriage, psychiatric illness, tobacco/alcohol/drug abuse in the family)

....

....

Nil Significant ☐

VIII. Epidemiological History

(History of recent travel, contact with similar cases (for communicable diseases), etc.)

....

....

Nil Significant ☐

IX. Socio-environmental History

(Overcrowding, lack of ventilation, source of drinking water, water purification methods used, etc.)

....

....

Nil Significant ☐

X. Personal history

a) Bowel

Not available ☐ N.A.D ☐

b) Bladder

Not Available ☐ N.A.D ☐

c) Sleep

Not Available ☐ N.A.D ☐

d) Appetite

Not Available ☐ N.A.D ☐

e) Addiction

Elaborate on the duration, type of addiction

Not Available ☐ Nil ☐

XI. General Physical Examination

External causes

Clothing

(Any evidence of violence, wetness, burns, any foreign substance)

Nil Significant ☐

Entire body

(Any evidence of injuries; describe the site, number, and nature of injuries; any foreign substance or bodily fluids/discharge)

Nil significant ☐

Eyes

(any hemorrhage)

Nil Significant ☐

Ears

(any hemorrhage, CSF in the canal)

Nil Significant ☐

Mouth

(Evidence of any foreign substance)

Nil Significant ☐

Routine

Pallor

(Elaborate on the site, and severity)

Nil Significant ☐

Icterus

(Elaborate on the site, and severity)

Nil Significant ☐

Cyanosis

(Elaborate on the site, severity, characteristics)

Nil Significant ☐

Clubbing

(Elaborate on the grade)

Nil Significant ☐

Lymphadenopathy

(Elaborate on distribution (Localized/generalized), site, characteristics)

Nil Significant ☐

Loss of subcutaneous fat

(Elaborate on distribution, severity)

Nil Significant ☐

Edema

(Elaborate on region involved, severity, nature)

Nil Significant ☐

Signs of Dehydration

(Elaborate on region involved, severity)

Nil Significant ☐

Vitals

BP: in mmHg — Not Available ☐

Pulse: in bpm — Not Available ☐

RR: in cycles/min — Not Available ☐

Temperature: in °F — Not Available ☐

XII. Systemic examination

a) **RS**

Not Available ☐ N.A.D ☐

b) CVS

Not Available ☐ N.A.D ☐

c) P/A

Not Available ☐ N.A.D ☐

d) CNS

Not Available ☐ N.A.D ☐

XIII. Investigations

(List the findings from relevant investigations that have been conducted. Additionally, order any other investigations that you believe are necessary to determine the cause of death)

Haematological investigations

Not Available ☐ N.A.D ☐

Renal Function Tests

Not Available ☐ N.A.D ☐

Liver Function Test s

Not Available ☐ N.A.D ☐

Serum Lipid Profile

Not Available ☐ N.A.D ☐

Radiological Investigations

Not Available ☐ N.A.D ☐

Others

Not Available ☐ N.A.D ☐

Autopsy

If Autopsy is done for the case then Form4 in sl no.XVII also needs to be filled

Not Available ☐ N.A.D ☐

XIV. Remarks/Narrative

(Please record the diagnosis and sequence of events from MCCD form, any other information, narrative from the kin of the deceased)

XV. Summary

(All responses recorded above will auto populate in the respective fields except those recorded as NAD, Nil significant or Not available)

**Serial No**	
**Name of the doctor collecting history**	
**Name of the Deceased**	
**Age of the deceased**	
**Sex of the deceased**	
**House address**	
**Occupation of the diseased**	
**Date of death**	
**Place of death**	
**Chief Complaints and Duration**	
**History of Presenting Illness**	
**Treatment received for current illness**	
**Past history**	
**Family history**	
**Epidemiological history**	
**Socio-environmental history**	
**Personal history**	
**General Physical Examination**	
**Systemic examination**	
**Investigations**	
**Remarks/ Narrative**	

XVI. FORM NO. 4 (To be completed by the Clinician attending to the case)

FORM NO. 4

(See Rule 7)

MEDICAL CERTIFICATE OF CAUSE OF DEATH

(Hospital in-patients. Not to be used for still births)

To be sent to Registrar along with Form No.2 (Death Report)

Name of the Hospital……………………………………………………………………………………………………

I hereby certify that the person whose particulars are given below died in the hospital in Ward No……………on………………………………

at…………A.M./P.M.

Name of the Deceased					For use of Statistical Office
Sex	Age at Death				
	If 1 year or more, age in Years	If less than 1 year, age in Months	If less than one month, age in Days	If less than one day, age in Hours	
1. Male<br>2. Female					

CAUSE OF DEATH		Interval between on set & death approx	
I			
Immediate Cause	(a) ……………………………………		
State the disease, injury or complication which caused death, not the mode of dying such as heart failure, asthenia, etc.	Due to (or as a consequences of)	…………………	………………
Antecedent Cause	(b) ……………………………………	………………………	………………
Morbid conditions, if any, giving rise to the above Cause, stating underlying conditions last	Due to (or as a consequences of)<br>(c) ……………………………………	………………………	………………
II			
Other significant conditions contributing to the death but not related to the disease or conditions causing it	……………………………………………………<br>……………………………………………………	……………………<br>……………………	……………<br>……………

<u>Manner of death</u> How did the injury occur?

1. Natural 2. Accident 3. Suicide 4.Homicide

5. Pending investigation

If deceased was a female, was pregnancy death associated with? 1. Yes 2. No

If yes, was there a delivery? 1.Yes 2.No.

Name and signature of the Medical Attendant certifying the cause of death

Date of verification……………………………………

XVII. FORM NO.4 (To be completed by the person performing the autopsy)

FORM NO. 4

(See Rule 7)

MEDICAL CERTIFICATE OF CAUSE OF DEATH

(Hospital in-patients. Not to be used for still births)

To be sent to Registrar along with Form No.2 (Death Report)

Name of the Hospital………

I hereby certify that the person whose particulars are given below died in the hospital in Ward No……………on……………………………

at…………A.M./P.M.

<table>
<tr><td colspan="5">Name of the Deceased</td><td>For use of Statistical Office</td></tr>
<tr><td rowspan="2">Sex</td><td colspan="4">Age at Death</td><td rowspan="3"></td></tr>
<tr><td>If 1 year or more,age in Years</td><td>If less than 1 year, age in Months</td><td>If less than one month, age in Days</td><td>If less than one day, age in Hours</td></tr>
<tr><td>1. Male
2. Female</td><td></td><td></td><td></td><td></td></tr>
<tr><td colspan="4">CAUSE OF DEATH</td><td>Interval between on set & death approx</td><td></td></tr>
<tr><td colspan="4">I</td><td></td><td></td></tr>
<tr><td colspan="2">Immediate Cause

State the disease, injury or complication which caused death, not the mode of dying such as heart failure, asthenia, etc.</td><td colspan="2">(a) ……………………………………

Due to (or as a consequences of)</td><td>………………</td><td>………………</td></tr>
<tr><td colspan="2">Antecedent Cause
Morbid conditions, if any, giving rise to the above Cause, stating underlying conditions last</td><td colspan="2">(b) ………………………………………
Due to (or as a consequences of)
(c) ………………………………………</td><td>………………………

………………………</td><td>………………

………………</td></tr>
<tr><td colspan="4">II</td><td></td><td></td></tr>
<tr><td colspan="2">Other significant conditions contributing to the death but not related to the disease or conditions causing it</td><td colspan="2">……………………………………………………
……………………………………………………</td><td>……………………
……………………</td><td>……………
……………</td></tr>
<tr><td colspan="3">Manner of death
1. Natural 2. Accident 3. Suicide 4.Homicide
5. Pending investigation</td><td colspan="3">How did the injury occur?</td></tr>
<tr><td colspan="6">If deceased was a female, was pregnancy death associated with? 1. Yes 2. No
If yes, was there a delivery? 1.Yes 2.No.</td></tr>
<tr><td colspan="6">Name and signature of the Medical Attendant certifying the cause of death
Date of verification…………………………………</td></tr>
</table>
