# Supplementary material for: Establishing a System for Medical Certification of Cause of Death for Noninstitutional Deaths in a Selected Area of Kolar District, Karnataka, India: Protocol for a Population-Based Feasibility and Validation Study
Source: JMIR Res Protoc. 2025 Aug 18;14:e72330. doi: 10.2196/72330 (PMC12402730; doi:10.2196/72330)
Supplement: Multimedia Appendix 3 [file resprot_v14i1e72330_app3.pdf]

Setting up of a system for Medical Certification of Cause of Death for non-institutional deaths in a selected area of a Taluk of Kolar district, Karnataka: feasibility and validity

Neonatal Death Questionnaire
(Birth to 4 weeks)

I. General Details

Date of the Interview:

Serial No	
Name of the doctor collecting history	
Name of the Deceased	
Name of the Mother of the deceased	
Sex of the deceased	
Age of the deceased (in days if ≥ 1 day and in competed hours if < 1 day)	
House address	
Date of death	
Time of death	Not Available ☐
Place of death	Home ☐<br>Others ☐<br>If others Specify below<br>☐
Name of the Hospital where treated/brought dead	

II. Respondent details

Sl No	Name	Relationship	Contact No
1.			
2.			
3.			

III. Chief Complaints and Duration

(Based on the selection of chief complaints, duration box will appear for that specific chief complaint)

Sl No	Complaint	Duration (specify in days or hours)
1.	Fever	

2.	**Feeding difficulties**	
3.	**Failure to gain weight/ Weight loss**	
4.	**Breathing Difficulty**	
5.	**Cough**	
6.	**Bluish discoloration of skin/peripheries**	
7.	**Excessive cry**	
8.	**Running nose/Rhinorrhea**	
9.	**Edema**	
10.	**Abdominal Tenderness**	
11.	**Abdominal distension**	
12.	**Loose stools**	
13.	**Blood in stools**	
14.	**Mucus in stools**	
15.	**Change in color of stools**	
16.	**Vomiting**	
17.	**Yellowish discoloration of eyes, palms, nailbeds**	
18.	**Loss of consciousness**	
19.	**Altered sensorium**	
20.	**Convulsions**	
21.	**Lethargy/excessive drowsiness**	
22.	**Drooling of saliva**	
23.	**Restlessness**	
24.	**Jitteriness (jerky movements)**	
25.	**Abnormal movements**	
26.	**Decreased movements/Weakness of limbs**	
27.	**Excessive Sweating**	
28.	**Night sweats**	
29.	**Decreased passage of urine**	
30.	**Dark colored urine**	
31.	**Bleeding from the mucosa**	
32.	**Bleeding from the skin**	
33.	**Bleeding from the Cord Stump**	
34.	**Pallor**	
35.	**Congenital Malformation of any part**	
36.	**Cold extremities**	
37.	**Skin Rashes**	
38.	**Bulging or raised fontanelle**	
39.	**Sunken fontanelle**	
40.	**Any external causes (like poisoning, road traffic accidents, etc.,) *<br>Yes ☐**	

	**No** ☐	
**41.**	**Others specify**	
**42.**	**Others specify**	
**43.**	**Others specify**	

a) *mandatory

b) if no other chief complaint is chosen then, at least one "others specify" shall be mandatorily recorded.

IV. History of Presenting Illness

(Based on the selection of chief complaints, details box will appear for that specific chief complaint)

Sl No	Complaint	Details
**1.**	**Fever**	Elaborate on nature, severity, aggravating, relieving factors, associated complaints
**2.**	**Feeding difficulties**	Elaborate on the nature, associated complaints
**3.**	**Failure to gain weight/ Weight loss**	Elaborate on the loss of weight/weight gain over duration, associated complaints
**4.**	**Breathing Difficulty**	Elaborate on the nature, associated complaints (intercostal suction, abnormal sounds produced), precipitating, aggravating and relieving factors
**5.**	**Cough**	Elaborate on the severity, frequency of cough, nature, sputum characteristics, precipitating, aggravating, relieving factors, associated complaints
**6.**	**Bluish discoloration of skin/peripheries**	Elaborate on the site, severity, characteristics, aggravating and relieving factors, associated complaints
**7.**	**Excessive cry**	Elaborate on the number of episodes, the duration of episodes, severity, associated complaints
**8.**	**Running nose/Rhinorrhea**	Elaborate on the severity and associated complaints
**9.**	**Edema**	Elaborate on region involved, severity, nature, precipitating, aggravating and relieving factors, progress, associated complaints
**10.**	**Abdominal Tenderness**	Elaborate on the site, radiation, nature of pain, aggravating and relieving factors, progress, associated complaints

11.	**Abdominal distension**	Elaborate on the severity, characteristics, precipitating factors, progress, associated complaints
12.	**Loose stools**	Elaborate on the no. of episodes, consistency, color, contents, smell, associated complaints
13.	**Blood in stools**	Elaborate on the no. of episodes, quantity of blood lost in each episode, precipitating factors, associated complaints
14.	**Mucus in stools**	Elaborate on the no. of episodes, quantity of mucus lost in each episode, precipitating factors, associated complaints
15.	**Change in color of stools**	Elaborate on the color, frequency, precipitating factors, associated complaints
16.	**Vomiting**	Elaborate on the number of episodes, nature, content of the vomitus, precipitating factors, associated complaints
17.	**Yellowish discoloration of eyes, palms, nailbeds**	Elaborate on the site, severity and associated complaints
18.	**Loss of consciousness**	Elaborate on the number of episodes, duration of episodes, precipitating factors, associated complaints
19.	**Altered sensorium**	Elaborate on the nature of the alteration (e.g., confusion, disorientation and drowsiness), precipitating factors, progress, associated complaints
20.	**Convulsions**	Elaborate on the parts of the body affected, nature, number of episodes, precipitating, aggravating, relieving factors, associated complaints
21.	**Lethargy/excessive drowsiness**	Elaborate on severity, precipitating and relieving factors, associated complaints
22.	**Drooling of saliva**	Elaborate on the nature (continuous/ intermittent), severity and associated complaints
23.	**Restlessness**	Elaborate on the precipitating factors, relieving factors, number of episodes and duration of episodes
24.	**Jitteriness (jerky movements)**	Elaborate on the precipitating factors, relieving factors, number of episodes and duration of episodes

25.	**Abnormal movements**	Elaborate on the number and duration of episodes, site, severity and characteristics
26.	**Decreased movements/ Weakness of limbs**	Elaborate on the site, severity, precipitating factors, progress, associated complaints
27.	**Excessive Sweating**	Elaborate on the site(s), precipitating factors, number and duration of episodes and associated complaints
28.	**Night sweats**	Elaborate on precipitating factors, number and duration of episodes and associated complaints
29.	**Decreased passage of urine**	Elaborate on the amount, frequency and associated complaints
30.	**Dark colored urine**	Elaborate on the color, amount, frequency and associated complaints
31.	**Bleeding from the mucosa**	Elaborate on the sites, severity, size, number of episodes, quantity of blood lost in each episode, precipitating factor(s)
32.	**Bleeding from the skin**	Elaborate on the sites, severity, size, number of episodes, quantity of blood lost in each episode, precipitating factor(s)
33.	**Bleeding from the Cord Stump**	Elaborate on the severity, size, number of episodes, quantity of blood lost in each episode, precipitating factor(s)
34.	**Pallor**	Elaborate on the site(s), severity, associated complaints
35.	**Congenital Malformation of any part**	Specify site, nature
36.	**Cold extremities**	Elaborate on the site, number and duration of episodes, associated complaints
37.	**Skin Rashes**	Elaborate on the number/density, characteristics, distribution, evolution, associated complaints
38.	**Bulging or raised fontanelle**	Elaborate on the severity and associated complaints
39.	**Sunken fontanelle**	Elaborate on the severity and associated complaints

**40.**	**Any external causes (like poisoning, road traffic accident etc.,)***<br><br>**Yes** ☐ **(If Yes, elaborate in the details box beside)**<br>**No** ☐	Elaborate on the circumstances, intent (accident, suicide, homicide etc.,), site, nature of injury and place of occurrence
**41.**	**Others specify**	Elaborate on the site, number of episodes, duration of episodes, severity, nature, precipitating factors, relieving factors, associated complaints
**42.**	**Others specify**	Elaborate on the site, number of episodes, duration of episodes, severity, nature, precipitating factors, relieving factors, associated complaints
**43.**	**Others specify**	Elaborate on the site, number of episodes, duration of episodes, severity, nature, precipitating factors, relieving factors, associated complaints

a) *mandatory

b) If no other chief complaint is chosen then, at least one "others specify" shall be made mandatorily recorded

V. Treatment received for the current illness

(Record the history of any treatment received for the current illness)

....

....

Nil ☐

VI. Antenatal History

Order	☐	Not Available	☐
Whether Multiple Pregnancy	☐	Not Available	☐
LMP	☐	Not Available	☐
EDD	☐	Not Available	☐
No. of ANCs	☐	Not Available	☐
Abnormal findings during ANC	☐	Not Available	☐
IFA tablets taken or not	☐	Not Available	☐
No. of TT taken	☐	Not Available	☐

Any disease/ events during antenatal period		Not Available ☐
Age of the mother at pregnancy		Not Available ☐

VII. Natal history

Date of delivery		Not Available ☐
Duration of gestation		Not Available ☐
Mode of delivery		Not Available ☐
Reason		Not Available ☐
Significant events (like prolonged labor, birth trauma)		Not Available ☐
Place of delivery		Not Available ☐
Person conducting delivery		Not Available ☐

VIII. Post Natal history

APGAR score		Not Available ☐
Birthweight		Not Available ☐
Prelacteal feeds		Not Available ☐
Application on the stumps if any		Not Available ☐
Feeding (exclusively breastfed or formula fed, frequency)		Not Available ☐
Sucking (good/poor/no sucking)		Not Available ☐
Vaccination details		Not Available ☐
Birth trauma		Not Available ☐
Any other significant events		Not Available ☐

IX. Previous obstetric history

(Record any previous obstetric history of the mother of deceased as per the given fields)

....

....

Nil Significant ☐

X. Family History

(Similar illnesses, any other communicable diseases, consanguineous marriage, psychiatric illness, tobacco/alcohol/drug abuse in the family)

....

....

Nil Significant ☐

XI. Epidemiological History

(History of recent travel, contact with similar cases (for communicable diseases), etc.)

....

....

Nil Significant ☐

XII. Socio-environmental History

(Overcrowding, lack of ventilation, source of drinking water, water purification methods used, etc.)

....

....

Nil Significant ☐

XIII. Personal history

a) Bowel:

Not available ☐ N.A.D ☐

b) Bladder:

Not Available ☐ N.A.D ☐

c) Sleep:

Not Available ☐ N.A.D ☐

d) Feeding:

Not Available ☐ N.A.D ☐

XIV. General Physical Examination

External causes

Clothing:

(Any evidence of violence, wetness, burns, any foreign substance)

Nil Significant ☐

Entire body:

(Any evidence of injuries; describe the site, number, and nature of injuries; any foreign substance or bodily fluids/discharge)

Nil significant ☐

Eyes

(Any hemorrhage)

Nil Significant ☐

Ears

(Any hemorrhage, CSF in the canal)

Nil Significant ☐

Mouth

(Evidence of any foreign substance):

Nil Significant ☐

Routine

Pallor

(Elaborate on the site, and severity)

Nil Significant ☐

Icterus

(Elaborate on the site, and severity)

Nil Significant ☐

Cyanosis

(Elaborate on the site, severity, characteristics)

Nil Significant ☐

Clubbing

(Elaborate on the grade)

Nil Significant ☐

Lymphadenopathy

(Elaborate on distribution (Localized/generalized), site, characteristics)

Nil Significant ☐

Loss of subcutaneous fat

(Elaborate on distribution, severity)

Nil Significant ☐

Edema

(Elaborate on region involved, severity, nature)

Nil Significant ☐

Signs of Dehydration

(Elaborate on region involved, severity)

Nil Significant ☐

Vitals

**BP**	in mmHg	Not Available ☐
**Pulse**	in bpm	Not Available ☐
**RR**	in cycles/min	Not Available ☐
**Temperature**	in °F	Not Available ☐

XV. Systemic examination

a) RS

Not Available ☐ N.A.D ☐

b) **CVS**

Not Available ☐ N.A.D ☐

c) **P/A**

Not Available ☐ N.A.D ☐

d) **CNS**

Not Available ☐ N.A.D ☐

XVI. Investigations

List the findings from relevant investigations that have been conducted. Additionally, order any other investigations that you believe are necessary to determine the cause of death.

Haematological investigations

Not Available ☐ N.A.D ☐

Renal Function Tests

Not Available ☐ N.A.D ☐

Liver Function Tests

Not Available ☐ N.A.D ☐

Serum Lipid Profile

Not Available ☐ N.A.D ☐

Radiological Investigations

Not Available ☐ N.A.D ☐

Others

Not Available ☐ N.A.D ☐

Autopsy Findings

If Autopsy is done for the case then Form 4 in serial no.XX also needs to be filled

Not Available ☐ N.A.D ☐

XVII. Remarks/Narrative

(Please record the diagnosis and sequence of events from MCCD form, any other information, narrative from the kin of the deceased)

XVIII. Summary

(All responses recorded above will auto populate in the respective fields except those recorded as NAD, Nil significant or Not available)

**Serial No**	
**Name of the doctor collecting history**	
**Name of the Deceased**	
**Name of the Mother**	
**Age of the deceased**	
**Sex of the deceased**	
**House address**	
**Date of death**	
**Place of death**	
**Chief Complaints and Duration**	
**History of Presenting Illness**	
**Treatment received for current illness**	
**Antenatal History**	
**Natal History**	
**Post Natal History**	
**Previous gynecological/obstetric history**	
**Family History**	
**Epidemiological History**	
**Socio-environmental History**	
**Personal history**	
**General Physical Examination of the body**	
**Systemic examination**	
**Investigations**	
**Remarks/ Narrative**	

XIX. FORM NO. 4 (To be completed by the Clinician attending to the case)

FORM NO. 4

(See Rule 7)

MEDICAL CERTIFICATE OF CAUSE OF DEATH

(Hospital in-patients. Not to be used for still births)

To be sent to Registrar along with Form No.2 (Death Report)

Name of the Hospital...

I hereby certify that the person whose particulars are given below died in the hospital in Ward No...............on....................................

at............A.M./P.M.

Name of the Deceased					For use of Statistical Office
Sex	Age at Death				
	If 1 year or more,age in Years	If less than 1 year, age in Months	If less than one month, age in Days	If less than one day, age in Hours	
1. Male<br>2. Female					
**CAUSE OF DEATH**				Interval between on set & death approx	
I					
Immediate Cause	(a)				
State the disease, injury or complication which caused death, not the mode of dying such as heart failure, asthenia, etc.	Due to (or as a consequences of)			....	....
Antecedent Cause	(b)			....	....
Morbid conditions, if any, giving rise to the above Cause, stating underlying conditions last	Due to (or as a consequences of)<br>(c)			....	....
II					
Other significant conditions contributing to the death but not related to the disease or conditions causing it	....<br>...			.....................<br>.....................	.....................<br>.....................

<u>Manner of death</u>

1. Natural 2. Accident 3. Suicide 4.Homicide

5. Pending investigation

How did the injury occur?

If deceased was a female, was pregnancy death associated with? 1. Yes 2. No

If yes, was there a delivery? 1.Yes 2.No.

Name and signature of the Medical Attendant certifying the cause of death

Date of verification..

XX. FORM NO.4 (To be completed by the person performing the autopsy)

FORM NO. 4

(See Rule 7)

MEDICAL CERTIFICATE OF CAUSE OF DEATH

(Hospital in-patients. Not to be used for still births)

To be sent to Registrar along with Form No.2 (Death Report)

Name of the Hospital...

I hereby certify that the person whose particulars are given below died in the hospital in Ward No...............on..................................

at............A.M./P.M.

Name of the Deceased					For use of Statistical Office
Sex	Age at Death				
	If 1 year or more, age in Years	If less than 1 year, age in Months	If less than one month, age in Days	If less than one day, age in Hours	
1. Male<br>2. Female					

CAUSE OF DEATH		Interval between on set & death approx	
I			
Immediate Cause	(a)		
State the disease, injury or complication which caused death, not the mode of dying such as heart failure, asthenia, etc.	Due to (or as a consequences of)	....	....
Antecedent Cause	(b)	....	....
Morbid conditions, if any, giving rise to the above Cause, stating underlying conditions last	Due to (or as a consequences of)<br>(c)	....	....
II			
Other significant conditions contributing to the death but not related to the disease or conditions causing it	....<br>..	.........................<br>.........................	..................<br>..................

Manner of death

1. Natural 2. Accident 3. Suicide 4. Homicide

5. Pending investigation

How did the injury occur?

If deceased was a female, was pregnancy death associated with? 1. Yes 2. No

If yes, was there a delivery? 1.Yes 2.No.

Name and signature of the Medical Attendant certifying the cause of death

Date of verification..
