# Supplementary material for: Establishing a System for Medical Certification of Cause of Death for Noninstitutional Deaths in a Selected Area of Kolar District, Karnataka, India: Protocol for a Population-Based Feasibility and Validation Study
Source: JMIR Res Protoc. 2025 Aug 18;14:e72330. doi: 10.2196/72330 (PMC12402730; doi:10.2196/72330)
Supplement: Multimedia Appendix 4 [file resprot_v14i1e72330_app4.pdf]

Setting up of a system for Medical Certification of Cause of Death for non-institutional deaths in a selected area of a Taluk of Kolar district, Karnataka: feasibility and validity

Child Death Questionnaire (>1 month to 14 years)

I. General Details

Date of the Interview:

**Serial No**	
**Name of the doctor collecting history**	
**Name of the Deceased**	
**Name of the Husband/Father of the deceased**	
**Sex of the deceased**	
**Age of the deceased (in years if ≥ 1 year and in completed months if <1 year)**	
**House address**	
**Date of death**	
**Time of death**	**Not Available** ☐
**Place of death**	**Home** ☐<br>**Others** ☐<br>**If others, Specify below** ☐
**Name of the Hospital where treated/ Brought dead**	

II. Respondent details

Sl No	Name	Relationship	Contact No
1.			
2.			
3.			

III. Chief Complaints and Duration

(Based on the selection of chief complaints, duration box will appear for that specific chief complaint)

Sl No	Complaint	Duration (specify in days and if < 1 day in hours)
1.	Fever	
2.	Decreased feeding	
3.	Failure to gain weight/ Weight loss	
4.	Lag in the milestones	
5.	Breathing Difficulty	
6.	Cough	
7.	Bluish discoloration of skin/peripheries	
8.	Easy fatigability	
9.	Chest pain	
10.	Cold extremities	
11.	Pain abdomen	
12.	Protruding abdomen	
13.	Loose stools	
14.	Blood in stools	
15.	Mucus in stools	
16.	Change in color of stools	
17.	Vomiting	
18.	Yellowish discoloration of eyes, palms, nailbeds	
19.	Loss of consciousness	
20.	Altered sensorium	
21.	Weakness of limb(s)	
22.	Stiff neck	
23.	Stiffness of whole body	
24.	Headache	
25.	Convulsions	
26.	Lethargy/excessive drowsiness	
27.	Decreased passage of urine	
28.	Dark colored urine	
29.	Swelling/Edema	
30.	Bleeding from the mucosa	
31.	Bleeding from the skin	
32.	Pallor	
33.	Congenital Malformation of any part	
34.	Bulging or raised fontanelle	
35.	Sunken fontanelle	
36.	Skin Rashes	
37.	Wound(s)	
38.	Lumps	
39.	Any external causes (like poisoning, road traffic accident etc.,)*<br>Yes ☐<br>No ☐	
40.	Others specify	

**41.**	**Others specify**	
**42.**	**Others specify**	

a) *mandatory

b) if no other chief complaint is chosen then, at least one “others specify” shall be mandatorily recorded

IV. History of Presenting Illness

(Based on the selection of chief complaints, details box will appear for that specific chief complaint)

Sl No	Complaint	Details
**1.**	**Fever**	Elaborate on nature, severity, aggravating, relieving factors, associated complaints
**2.**	**Decreased feeding**	Elaborate on the sucking, associated complaints
**3.**	**Failure to gain weight/ Weight loss**	Elaborate on the loss of weight/weight gain over duration, associated complaints
**4.**	**Lag in the milestone(s)**	Elaborate on the type(s) of milestone(s), associated complaints
**5.**	**Breathing Difficulty**	Elaborate on associated manifestations (intercostal suction, abnormal sounds produced), associated complaints
**6.**	**Cough**	Elaborate on the severity, frequency of cough, nature, sputum characteristics, aggravating /relieving factors, associated complaints
**7.**	**Bluish discoloration of skin/peripheries**	Elaborate on the site, severity, characteristics, aggravating and relieving factors, associated complaints
**8.**	**Easy fatigability**	Elaborate on number of episodes, severity, progress, associated complaints
**9.**	**Chest pain**	Elaborate on the number of episodes, duration of episodes, severity, characteristics, radiation, associated complaints
**10.**	**Cold extremities**	Elaborate on the site, number and duration of episodes, associated complaints

**11.**	**Pain abdomen**	Elaborate on the site, radiation, nature of pain, precipitating, aggravating and relieving factors, progress, associated complaints
**12.**	**Abdominal distension**	Elaborate on the severity, characteristics, precipitating factors, progress, associated complaints
**13.**	**Loose stools**	Elaborate on the number of episodes, consistency, color, contents, smell, associated complaints
**14.**	**Blood in stools**	Elaborate on the number of episodes, quantity of blood lost in each episode, precipitating factors, associated complaints
**15.**	**Mucus in stools**	Elaborate on the number of episodes, quantity of mucus lost in each episode, precipitating factors, associated complaints
**16.**	**Change in color of stools**	Elaborate on the color, frequency, precipitating factors, associated complaints
**17.**	**Vomiting**	Elaborate on the number of episodes, nature, content of the vomitus, precipitating factors, associated complaints
**18.**	**Yellowish discoloration of eyes, palms, nailbeds**	Elaborate on the site, severity, associated complaints
**19.**	**Loss of consciousness**	Elaborate on the number of episodes, duration of episodes, precipitating factors, associated complaints
**20.**	**Altered sensorium**	Elaborate on the number of episodes, duration of episodes, severity, nature ((e.g., dementia, hallucinations, confusion, disorientation etc.,), precipitating factor(s), progress and associated complaints
**21.**	**Weakness of limb(s)**	Elaborate on the site, precipitating factor(s), progress, associated complaints
**22.**	**Stiff neck**	Elaborate on severity, precipitating factor(s), associated complaints
**23.**	**Stiffness of other body parts**	Elaborate on site, precipitating factor(s), severity, associated complaints

**24.**	**Headache**	Elaborate on the nature, severity, precipitating, aggravating and relieving factors, associated complaints
**25.**	**Convulsions**	Elaborate on the nature, parts of the body affected, number of episodes, precipitating, aggravating, relieving factors, associated complaints
**26.**	**Lethargy/excessive drowsiness**	Elaborate on the precipitating factor(s), relieving factors, number and duration of episodes
**27.**	**Decreased passage of urine**	Elaborate on the amount, frequency, associated complaints
**28.**	**Dark colored urine**	Elaborate on the color, amount, frequency, associated complaints
**29.**	**Swelling/Edema**	Elaborate on region involved, severity, characteristics, precipitating, aggravating and relieving factors, progress, associated complaints
**30.**	**Bleeding from the mucosa**	Elaborate on the site, severity, size, number of episodes, quantity of blood lost in each episode, precipitating factor(s), associated complaints
**31.**	**Bleeding from the skin**	Elaborate on the site, severity, size, number of episodes, quantity of blood lost in each episode, precipitating factor(s), associated complaints
**32.**	**Pallor**	Elaborate on the site, severity, associated complaints
**33.**	**Congenital Malformation of any part**	Specify site, nature
**34.**	**Bulging or raised fontanelle**	Elaborate on the severity, associated complaints
**35.**	**Sunken fontanelle**	Elaborate on the severity, associated complaints
**36.**	**Skin Rashes**	Elaborate on the number/density, characteristics, distribution, evolution, associated complaints
**37.**	**Wounds**	Elaborate on the site, extent, number, nature, precipitating factor, progress, associated complaints

**38.**	**Lumps**	Elaborate on the size, distribution, site, characteristics, precipitating factor(s), progress, associated complaints
**39.**	**Any external causes (like poisoning, road traffic accident etc.,)***<br>**Yes** ☐ **(If Yes, elaborate in the details box beside)**<br>**No** ☐	Elaborate on the circumstances, intent (accident, suicide, homicide etc.,), site and nature of injury and place of occurrence
**40.**	**Others specify**	Elaborate on the site, number of episodes, duration of episodes, severity, nature, precipitating factors, relieving factors, associated complaints
**41.**	**Others specify**	Elaborate on the site, number of episodes, duration of episodes, severity, nature, precipitating factors, relieving factors, associated complaints
**42.**	**Others specify**	Elaborate on the site, number of episodes, duration of episodes, severity, nature, precipitating factors, relieving factors, associated complaints

a) *mandatory
b) If no other chief complaint is chosen then, at least one “others specify “shall be mandatorily recorded

V. Treatment received for the current illness

(Record the history of any treatment received for the current illness)

……

……

Nil Significant ☐

VI. Past History

(Please record duration and treatment received for all the selected diseases. if your selection is “Major Surgeries” then please specify the type of surgery performed and also the duration since the surgery)

Underweight

Elaborate on the degree and treatment taken

Stunting

Elaborate on the degree and treatment taken

Tuberculosis	Elaborate on duration and treatment taken
Epilepsy	Elaborate on duration and treatment taken
Bronchial asthma	Elaborate on duration and treatment taken
Covid-19	Elaborate on duration and treatment taken
Others	Elaborate on duration and treatment taken
Major surgeries	Specify the type and the duration since the surgery

VI. Antenatal history/Natal history

Obstetric score of the mother		Not available	☐
LMP		Not available	☐
Whether multiple pregnancy		Not available	☐
Abnormal findings during AN checkup if any		Not available	☐
IFA tabs		Not available	☐
TT taken		Not available	☐

Any disease/events during antenatal period		Not available ☐
Age of mother at pregnancy		Not available ☐
Duration of gestation		Not available ☐
Mode of delivery		Not available ☐
Reason		Not available ☐
Significant events (like prolonged labor, birth trauma etc)		Not available ☐
Place of delivery		Not available ☐
Person conducting delivery		Not available ☐

VII. Post Natal history

APGAR score		Not available ☐
Birthweight		Not available ☐
Prelacteal feeds		Not available ☐
Application on the stumps if any		Not available ☐
Feeding (exclusively breast fed or formula fed)		Not available ☐
Sucking (good/poor/no sucking)		Not available ☐
Vaccination status		Not available ☐
Any other significant event(s)		Not available ☐

VIII. Developmental history

(Elaborate on whether the milestones attained were on par with other siblings/ children of relatives or delayed)

Physical

Not available ☐ NAD ☐

Mental/ Emotional

Not available ☐ NAD ☐

IX. Family History

(Of similar illnesses, any other communicable diseases, consanguineous marriage, psychiatric illness/drug abuse in the family)

....

....

Nil significant ☐

X. Epidemiological history

(History of recent travel, contact with similar cases (for communicable diseases) etc.,

....

....

Nil significant ☐

XI. Socio environmental history

(Overcrowding, lack of ventilation, source of drinking water, water purification methods used etc.,)

....

....

Nil significant ☐

XII. Personal history

a) **Bowel**:

Not available ☐ N.A.D ☐

b) **Bladder**

Not Available ☐ N.A.D ☐

c) **Sleep**

Not Available ☐ N.A.D ☐

d) **Addiction**

Elaborate on the duration, type of addiction

Not Available ☐ N.A.D ☐

XIII. General Physical Examination

External causes

Clothing:

(Any evidence of violence, wetness, burns, any foreign substance)

Nil Significant ☐

Entire body

(Any evidence of injuries; describe the site, number, and nature of injuries; any foreign substance or bodily fluids/discharge)

Nil significant ☐

Eyes

(Any hemorrhage)

Nil Significant ☐

Ears

(Any hemorrhage, CSF in the canal)

Nil Significant ☐

Mouth

(Evidence of any foreign substance)

Nil Significant ☐

Routine

Pallor

(Elaborate on the site, and severity)

Nil Significant ☐

Icterus

(Elaborate on the site, and severity)

Nil Significant ☐

Cyanosis

(Elaborate on the site, severity, characteristics)

Nil Significant ☐

Clubbing

(Elaborate on the grade)

Nil Significant ☐

Lymphadenopathy

(Elaborate on distribution (Localized/generalized), site, characteristics)

Nil Significant ☐

Loss of subcutaneous fat

(Elaborate on distribution, severity)

Nil Significant ☐

Edema

(Elaborate on region involved, severity, nature)

Nil Significant ☐

Signs of Dehydration

(Elaborate on sign(s), severity)

Nil Significant ☐

Vitals

BP in mmHg Not Available ☐

Pulse in bpm Not Available ☐

RR in cycles/min — Not Available ☐

Temperature in °F — Not Available ☐

XIV. Systemic examination

a) **RS**

Not Available ☐ N.A.D ☐

b) CVS

Not Available ☐ N.A.D ☐

c) P/A

Not Available ☐ N.A.D ☐

d) CNS

Not Available ☐ N.A.D ☐

XV. Investigations

(List the findings from relevant investigations that have been conducted. Additionally, order any other investigations that you believe are necessary to determine the cause of death)

Haematological investigations

Not Available ☐ N.A.D ☐

Renal Function Tests

Not Available ☐ N.A.D ☐

Liver Function Tests

Not Available ☐ N.A.D ☐

Serum Lipid Profile

Not Available ☐ N.A.D ☐

Radiological Investigations

Not Available ☐ N.A.D ☐

Others

Not Available ☐ N.A.D ☐

Autopsy Findings

If Autopsy is done for the case then Form 4 in serial no.XIX needs to be filled

Not Available ☐ N.A.D ☐

XVI. Remarks/ Narrative

(Please include diagnosis and sequence of events from MCCD form if available, any other information, narrative from the kin of the deceased)

XVII. Summary

(All responses recorded above will auto populate in the respective fields except those recorded as NAD, Nil significant or Not available)

**Serial No**	
**Name of the doctor collecting history**	
**Name of the Deceased**	
**Name of the Mother**	
**Age of the deceased**	
**Sex of the deceased**	
**House address**	
**Date of death**	
**Place of death**	
**Chief Complaints and Duration**	
**History of Presenting Illness**	
**Treatment received for the current illness**	
**Past History**	
**Antenatal /Natal History**	
**Post Natal History**	
**Developmental History**	
**Family History**	
**Epidemiological History**	
**Socio-environmental History**	
**Personal history**	
**General Physical Examination**	
**Systemic examination**	
**Investigations**	
**Remarks/ Narrative**	

XVIII. Form no 4 (To be completed by the Clinician attending to the case)

FORM NO. 4

(See Rule 7)

MEDICAL CERTIFICATE OF CAUSE OF DEATH

(Hospital in-patients. Not to be used for still births)

To be sent to Registrar along with Form No.2 (Death Report)

Name of the Hospital……………………………………………………………………………

I hereby certify that the person whose particulars are given below died in the hospital in Ward No……………on………………………………

at…………A.M./P.M.

Name of the Deceased					For use of Statistical Office
Sex	Age at Death				
	If 1 year or more, age in Years	If less than 1 year, age in Months	If less than one month, age in Days	If less than one day, age in Hours	
1. Male<br>2. Female					

CAUSE OF DEATH		Interval between on set & death approx	
I			
Immediate Cause	(a) ………………………………		
State the disease, injury or complication which caused death, not the mode of dying such as heart failure, asthenia, etc.	Due to (or as a consequences of)	………………	………………
Antecedent Cause	(b) ………………………………	………………	………………
Morbid conditions, if any, giving rise to the above Cause, stating underlying conditions last	Due to (or as a consequences of)<br>(c) ………………………………	………………	………………
II			
Other significant conditions contributing to the death but not related to the disease or conditions causing it	……………………………………<br>……………………………………	………………<br>………………	………………<br>………………

<u>Manner of death</u>

1. Natural 2. Accident 3. Suicide 4.Homicide
5. Pending investigation

How did the injury occur?

If deceased was a female, was pregnancy death associated with? 1. Yes 2. No

If yes, was there a delivery? 1.Yes 2.No.

Name and signature of the Medical Attendant certifying the cause of death

Date of verification……………………………………

XIX. Form no 4 (To be completed by the person performing the autopsy)

FORM NO. 4

(See Rule 7)

MEDICAL CERTIFICATE OF CAUSE OF DEATH

(Hospital in-patients. Not to be used for still births)

To be sent to Registrar along with Form No.2 (Death Report)

Name of the Hospital...

I hereby certify that the person whose particulars are given below died in the hospital in Ward No...............on.................................

at............A.M./P.M.

Name of the Deceased					For use of Statistical Office
Sex	Age at Death				
	If 1 year or more, age in Years	If less than 1 year, age in Months	If less than one month, age in Days	If less than one day, age in Hours	
1. Male<br>2. Female					

CAUSE OF DEATH		Interval between on set & death approx	
I			
Immediate Cause	(a)		
State the disease, injury or complication which caused death, not the mode of dying such as heart failure, asthenia, etc.	Due to (or as a consequences of)	....	....
Antecedent Cause	(b)	....	....
Morbid conditions, if any, giving rise to the above Cause, stating underlying conditions last	Due to (or as a consequences of)<br>(c)	....	....
II			
Other significant conditions contributing to the death but not related to the disease or conditions causing it	....<br>..	.............................<br>.............................	....................<br>....................

Manner of death

1. Natural 2. Accident 3. Suicide 4.Homicide

5. Pending investigation

How did the injury occur?

If deceased was a female, was pregnancy death associated with? 1. Yes 2. No

If yes, was there a delivery? 1.Yes 2.No.

Name and signature of the Medical Attendant certifying the cause of death

Date of verification..

Date of verification..
